# Missed Abortion Presented with Worsening Hyperemesis Gravidarum

**DOI:** 10.7759/cureus.7499

**Published:** 2020-04-01

**Authors:** Kulachanya Suwanwongse, Nehad Shabarek

**Affiliations:** 1 Internal Medicine, Lincoln Medical Center, New York City, USA

**Keywords:** abort, hyperemesis gravidarum, pregnancy, case report, high-risk obstetrics

## Abstract

Hyperemesis gravidarum (HG) is the most common cause of in-patient hospitalizations during the first half of pregnancy. The etiology of HG has not yet been elucidated, and the treatment is mainly symptomatic. Untreated severe HG can lead to catastrophic maternal complications such as cardiac arrhythmia and death. In contrast, the impact of untreated severe HG on the fetuses remains contradictory. Evidence suggested that HG may increase the risk of a small for gestational age (GA) fetus. We here report a case of 32-year-old nulliparous woman, GA of 14 weeks, who presented with worsening HG and later had a diagnosis of missed abortion. More research is needed to clarify the possibility of HG as a contributory cause of abortion.

## Introduction

Hyperemesis gravidarum (HG) accounts for the highest number of in-patient hospitalizations during the first half of pregnancy [1]. The pathogenesis of HG remains unclear so that the treatment is mainly supportive. HG can lead to several serious maternal complications, including severe dehydration, nutritional deficiency, and multiple electrolytes imbalances, which can result in cardiac arrhythmia and death [2]. However, HG-related fetal complications remain contradictory. Some studies suggested that HG causes abnormal placentation leading to pre-eclampsia and small for gestational age (GA) of the fetus [3]. To date, there is no conclusive evidence support whether HG directly increases the risk of abortion. In fact, patients with abortion are likely to have resolving HG due to a drop in gestational hormones. We report a unique case of worsening HG as a presentation of a missed abortion.

## Case presentation

**Figure 1:**
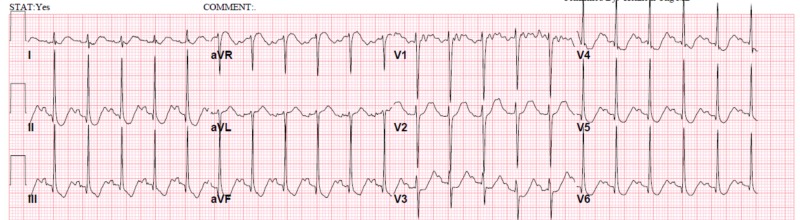
Electrocardiogram showed sinus tachycardia with a heart rate of 135 beats per minute and marked ST abnormalities

A 32-year-old nulliparous woman with GA of 14 weeks came to the hospital due to worsening nausea and vomiting for two days. She had had nausea and vomiting for approximately a month, without apparent causes, which was diagnosed as HG and received symptomatic treatment. She had no other medical problems nor any other complaints. On initial evaluation, she had tachycardia and significant dehydration without fever. Her blood tests were abnormal for hyponatremia with sodium of 131 millimoles per liter (mmol/L), hypokalemia with potassium of 2.8 mmol/L, and hypophosphatemia with phosphorus of less than 0.3 mmol/L. She also had elevated creatinine at 1.1 milligrams per deciliter (mg/dl) (her baseline creatinine was 0.52 mg/dl) and metabolic acidosis with an anion gap of 32 due to starvation ketoacidosis. Her electrocardiogram (ECG) showed sinus tachycardia with a heart rate of 135 beats per minute and marked ST abnormalities (Figure 1).

**Figure 2:**
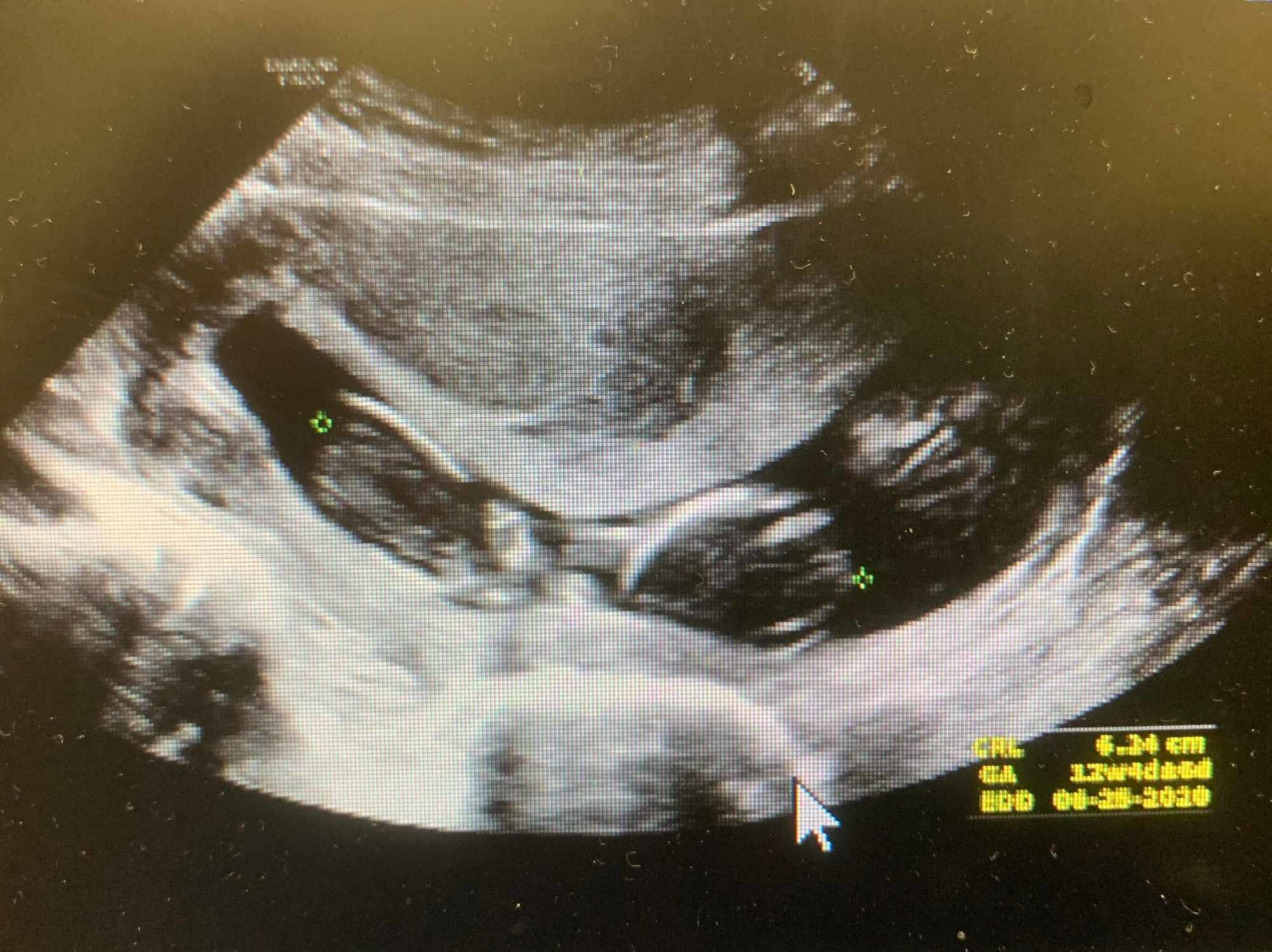
Ultrasound pelvis found an abnormal appearing fetus without a cardiac activity, estimated GA of 12 weeks, and four days GA, gestational age.

Ultrasound pelvis illustrated an abnormal appearing fetus without a cardiac activity, estimated GA of 12 weeks, and four days (Figure 2), compared to the previous ultrasound study a month prior, showed GA of 10 weeks and one day, indicating an interval missed abortion.

She was admitted to a medical intensive care unit, received aggressive electrolyte replacement and intravenous crystalloid bolus, and underwent dilatation and evacuation to remove the conceptus. Her postoperative course was uneventful. Her hospital course was complicated by an adjustment disorder with depressed mood, which was managed by supportive psychotherapy. Her conditions were improved, and she was discharged home on day 5.

## Discussion

HG is one of the most devastating forms of nausea and vomiting during pregnancy, leading to serious maternal morbidity and death [2]. However, HG related to adverse fetal outcomes remains controversial. HG may result in low birth weight and pre-eclampsia [3,4]. We presented a case of severe HG, leading to several critical electrolytes abnormalities, significant dehydration, and acute kidney injury. In our patient, prompt recognition and treatment of HG-related complications leads to favorable outcomes and reduces hospital length of stay. HG-related maternal complications requires urgent management to prevent catastrophic outcomes, such as cardiac arrhythmia, renal failure, nutritional deficiency, and death.

Patients suffering from HG presented with frequent vomiting and unable to tolerate oral intake, which will result in dehydration, metabolic alkalosis, electrolyte abnormalities, and nutritional deficits. These might result in life-threatening cardiac arrhythmias, seizures, acute kidney injury, osmotic demyelination syndrome, and Wernicke encephalopathy. Severe vomiting can also lead to fatal esophageal rupture and pneumomediastinum [5,6]. 

The differential diagnosis of HG includes gastrointestinal tract disorders, hepatobiliary diseases, pyelonephritis, and endocrine abnormalities. HG is a diagnosis of exclusion without any valid diagnostic markers. The treatment of HG is mainly supportive: the replacement of fluid and electrolytes and administration of antiemetic medications. 

Our patient was also found to have missed abortion, which may point out that severe HG can lead to fetal death and abortion. However, more evidence is needed to support our hypothesis.

## Conclusions

HG is a common and potentially lethal condition during pregnancy. Clinicians should be aware of HG-related complications. Our case report presentation suggested that severe HG may also result in abortion.
